# The Association Between Leucine and Diabetic Retinopathy in Different Genders: A Cross-Sectional Study in Chinese Patients With Type 2 Diabetes

**DOI:** 10.3389/fendo.2022.806807

**Published:** 2022-03-07

**Authors:** Shen Li, Bing Huang, Ming-Li Liu, Xue-Ting Cui, Yun-Feng Cao, Zheng-Nan Gao

**Affiliations:** ^1^Department of Endocrinology, Dalian Municipal Central Hospital, Dalian, China; ^2^Department of Science, Dalian Runsheng Kangtai Medical Lab Co. Ltd., Dalian, China; ^3^Shanghai Institute for Biomedical and Pharmaceutical Technologies, NHC Key Laboratory of Reproduction Regulation, Shanghai Engineering Research Center of Reproductive Health Drug and Devices, Shanghai, China; ^4^Dalian Institute of Chemical Physics, Chinese Academy of Sciences, Dalian, China

**Keywords:** leucine, diabetic retinopathy, gender, type 2 diabetes, cross-sectional study

## Abstract

**Objective:**

To explore the association between serum leucine (leu) and diabetic retinopathy (DR) in patients with type 2 diabetes (T2D) and then to analyze the influence of gender on the association.

**Method:**

The electronic medical records of 1,149 T2D patients who met inclusion and exclusion criteria were retrieved from the Second Affiliated Hospital of Dalian Medical University and the First Affiliated Hospital of Jinzhou Medical University. Serum leu levels of all subjects were measured by liquid chromatography–mass spectrometry. Logistic regression was used to obtain the odds ratio (OR) and CI of leu–DR risk in multiple models. When using these models, restricted cubic spline (RCS) was used to test the potential non-linear relationship between multiple continuous independent variables, such as leu and DR (classification), and dependent variables. We also used the additive interaction method to evaluate the interaction effect between leu and gender on DR.

**Results:**

Leu was a protective factor of DR [0.78 (0.66, 0.92)]. When gender was divided into male and female, the above relationship was statistically significant only in men [0.73 (0.58, 0.94)]. Three indicators of additive interaction—RERI, AP, and S—suggested that there is no interaction between gender and leu on the risk of DR.

**Conclusions:**

Male T2D patients with high leu levels may have a lower risk of DR.

## Introduction

Diabetes is a typical metabolic disease with high prevalence and serious complications (1), and the number of people suffering from the disease is increasing at an accelerated rate. In 2012, statistics showed that the number of people with the disease had reached 370 million, and it is expected that 552 million people worldwide will be affected by diabetes by 2030 (2). Due to a lack of awareness of diabetes screening, a significant number of patients only seek medical attention after the onset of symptoms, and the chronic nature of the disease leads to a lack of attention, which ultimately results in a higher and higher rate of complications and increasingly severe symptoms. In addition to increasing the risk of common cardiovascular and cerebrovascular diseases, it can also lead to microvascular diseases such as diabetic retinopathy (DR) and diabetic nephropathy (DN) (3). Among the microvascular diseases, DR is common and serious with a prevalence rate of 8.1% in diabetic patients in China (4), which can lead to irreversible blindness and bring a heavy burden to patients and their families. However, the prevalence of DR in China is significantly lower than in the rest of the world. Even after adjusting for confounding factors such as age–sex composition, the prevalence of DR in China (19.9%) remains lower than in the world (34.6%), Norway (34.6%), the United States (28.5%), Iceland (25.2%), and Africa (30.2%–31.6%) (5–9). The above data suggest that DR deserves global attention and exploration.

In recent years, metabolomics techniques have gradually become a new approach to the study of diabetes, providing a more scientific basis for the prevention and treatment of diabetes and its complications (10–12). Studies have found that branched-chain amino acids (BACC: leucine (leu), isoleucine, and valine) show an increasing trend in patients with metabolic syndrome (13–15), implying that elevated serum BACC are associated with the development of insulin resistance. BACC are very important signaling molecules in human anabolism, of which leu is involved in protein synthesis and is also a potent activator of mammalian target of rapamycin (mTOR), a serine/threonine kinase involved in many cellular processes, including protein synthesis, cell growth, and metabolism (16–19). Indeed, supplementation with leu increases net protein metabolism in a variety of tissues (20, 21). However, by activating mTOR and S6 kinase, leu also feedback-inhibits insulin signaling and reduces glucose utilization in skeletal muscle (22, 23), which means that leu is likely to be a new marker like body mass index (BMI), which can help identify patients with metabolic syndrome (13, 14, 24–27). It is now widely accepted that leu is involved in the development and progression of diabetes (28–32), but relatively little research has been done on its complications in DR. We know that when blood sugar is better controlled, the incidence and severity of associated complications can be improved (33, 34). We can therefore assume that the use of leu can improve blood glucose while reducing the severity of DR, and they act by the same mechanism.

In view of this, case reports of 1,149 patients with type 2 diabetes (T2D) from the Second Affiliated Hospital of Dalian Medical University and the First Affiliated Hospital of Jinzhou Medical University were collected in this paper to further explore the association between serum leu concentration and DR in patients with T2D and to analyze the influence of gender on the above association.

## Materials and Methods

### Study Method and Population

The electronic medical records of 1,306 patients with diabetes were retrieved from the Second Affiliated Hospital of Dalian Medical University (Liaoning, China) and the First Affiliated Hospital of Jinzhou Medical University (Liaoning, China). The inclusion criteria were individuals diagnosed with T2D; the exclusion criteria were 1) age under 18 years and 2) absence of any indicators of concern. Of the 1,306 patients, 1,286 were identified with T2D; 1,285 of them were older than 18 years; 1,149 patients were retained after final deletion due to partial absence of triglyceride (TG), high-density lipoprotein cholesterol (HDL_C), low-density lipoprotein cholesterol (LDL_C), serum creatinine (SCR), estimated glomerular filtration rate (eGFR), urinary creatinine (UA), and leu, of which 269 (23.41%) were diagnosed with DR. The diagnosis and differentiation of T2D patients are based on the relevant standards of WHO (2011) diabetes etiology Credit System (35). The diagnosis and identification of retinal lesions are based on “the new Edition of the Guidelines for the Diagnosis and Treatment of Diabetic Retinopathy and Macular Edema” (36), as revised by the Royal College of Ophthalmologists (RCM) in 2020.

The Ethics Committee for Clinical Research of the Second Affiliated Hospital of Dalian Medical University and the First Affiliated Hospital of Jinzhou Medical University approved the ethics of the study, and informed consent was waived due to the retrospective nature of the study, which is consistent with the Declaration of Helsinki.

### Data Collection and Clinical Definitions

Through consulting data and consulting doctors, 20 indices including baseline data, laboratory test indices, and complications of patients with T2D were determined and retrieved from the electronic medical records of the Second Affiliated Hospital of Dalian Medical University and the First Affiliated Hospital of Jinzhou Medical University. Baseline data included age, gender, BMI, smoke, drink, systolic blood pressure (SBP), diastolic blood pressure (DBP), and course of the disease (time). The laboratory indicators included Val (valine), glycosylated hemoglobin (HbA1c), TG, HDL_C, low-LDL_C, SCR, eGFR, and UA. Complications included DR, DN, stroke, and coronary heart disease (CHD). Among them, gender, smoking, alcohol consumption, DN, DR, stroke, and CHD were 7 indicator classification variables, and the remaining 13 indicators were continuous variables.

Anthropometric indices were measured by using standardized procedures in hospitals. Participants were allowed to wear light clothes and no shoes; height and weight were measured to the nearest 0.5 cm and 0.1, kg respectively. Blood pressure was measured behind the right arm by an adult cuff using a standard mercury sphygmomanometer and post-measurement at an appropriate size, after a 10-min rest while in a sitting position. Age was calculated from the date of birth to the date of hospitalization or medical examination and was calculated in years. The BMI was calculated as the ratio of weight (kg) to squared height (m) classifying overweight and obesity according to the criteria (37) recommended by the National Health Commission in China. The identification of DR is usually based on the patient’s diabetes history combined with ophthalmologic fundus examination and special examination (fundus fluorescence angiography). Microaneurysms and small hemorrhagic spots are the earliest and relatively accurate signs of retinopathy. The severity of DR was not classified in this paper.

### Laboratory Assay

Dried blood spots were used in the assay of metabolomics, which were prepared from capillary whole blood through 8-h fasting. The metabolites were measured by direct infusion mass spectrometry technology equipped with the AB Sciex 4000 QTrap system (AB Sciex, Framingham, MA, USA). High-purity water and acetonitrile were purchased from Thermo Fisher (Waltham, MA, USA) and utilized as diluting agent and mobile phase. 1-Butanol and acetyl chloride were obtained from Sigma-Aldrich (St Louis, MO, USA). Isotope-labeled internal standard samples of amino acids (NSK-A) were purchased from Cambridge Isotope Laboratories (Tewksbury, MA, USA), while standard samples of the leu were purchased from Chromsystems (Grafelfing, Germany). That is to say, 8.5 ml of venous blood was drawn from each participant at 0800 to 0930h after 8-h fasting. Laboratory tests were carried out at a special diagnostic laboratory. The level of lipid profiles was analyzed by an automatic biochemistry analyzer (Hitachi 7150, Tokyo, Japan). The levels of HDL-C and LDL-C were also assessed by the selective solubilization method (Determiner L-HDL, LDL test kit; Kyowa Medex, Tokyo, Japan).

### Statistical Analysis

Continuous variables are described by mean ± SD when they meet the normal distribution and median ± quartile spacing (interquartile range) if otherwise. The categorical variable was in numbers (percentage). When comparing whether there are differences in continuous variables between patients with DR and non-patients (N-DR), if the normal distribution is satisfied, a two-independent-samples t-test is used. On this condition, if homogeneity of variance is not satisfied, the Satterthwaite method will be used to adjust the degrees of freedom. If the normal distribution is not satisfied, the rank-sum test is used. The chi-square test was used to compare whether there were differences in classification variables between patients with DR and N-DR. Whether there was a difference between the DR group and N-DR groups was tested separately in men and women.

A binary logistic regression model was established to obtain the odds ratio (OR) and their 95% CIs. Traditional risk factors for T2D patients with DR were adjusted using a structured adjustment scheme. In this paper, 8 models were constructed to explore the relationship between leu and DR. Leu was incorporated into the model according to classification variables and continuous variables (2 categories in total). leu, leu + baseline data (Model2), leu + baseline data + laboratory test index (Model3), and leu + baseline data + laboratory test index + complication index (Model4) (4 categories in total) were used to construct 8 models (8 = 2 * 4). A restricted cubic spline (RCS) curve is a smooth curve, which is often used when the linear relationship between independent variables and dependent variables is not satisfied in a model. According to the sample size, 5-node RCS was chosen (the number of nodes has little influence on fitting, and the location is more important). By observing RCS curves and combining professional knowledge, the last point of leu and DR curves when OR = 1 was selected as the cutoff point.

Repeated logistic regression analysis was performed in men and women to obtain OR values. Additive interaction analysis was used to verify the relationship between gender (male or female) and leu (in 2 groups by RCS cutoff) for DR (38, 39). The relative excess risk due to interaction (RERI), attributable proportion due to interaction (AP), and synergy index (S) were calculated to estimate additive interactions (40). RERI > 0, AP > 0, or S > 1 indicates a significant additive interaction.

Of 1,149 samples, 80% were randomly selected as a training set to build a logistic regression model. The remaining 20% (N = 229) serve as a validation set to check the merits of the constructed model. At the same time, the variable leu (Model5) was removed on the basis of Model4, and finally, four receiver operating characteristic (ROC) curves (Model4 and Model5) * (with leu and without leu) were drawn to verify the validity of the model and the contribution of leu in the model (leu is included in the form of categorical variables).

The significance level was 0.05, which means p < 0.05 was considered statistically significant. All analyses were performed using R version 4.1.1 and SAS version 9.4 (Institute Inc., Cary, NC, USA).

## Result

### Description of Study Subjects

A total of 1,149 patients with T2D were collected in this study, including 558 (48.56%) women. A total of 269 diabetic patients were diagnosed with DR, including 126 (46.84%) men. The mean age and BMI of all patients were 62.00 (54.00–68.00) and 25.91 (23.51–28.37). **Table 1** shows the baseline data, laboratory test indicators, and complications of the patients based on gender, and the results showed the following:

1. The results were consistent when leu was analyzed as a continuous variable or a categorical variable, indicating that it was a protective factor in all genders and men, while the difference was not statistically significant in women.
2. Age (continuity) was a risk factor for DR in all genders. After stratification by gender, these relationships were found only in men. Age was no longer a risk factor for retinopathy after stratification at 65 years, and there was no difference between genders.
3. When HDL_C was analyzed as a continuous variable, baseline information HDL_C was a protective factor for DR in both men and women. When analyzed by categorical variables, HDL_C was a protective factor for DR in men, the percentage of men with DR who had high levels of HDL_C was significantly lower, and there exists no statistically significant difference among women.
4. Baseline information LDL_C was a risk factor for DR in both men and women when LDL_C was analyzed by continuous variables and categorical variables. The proportion of DR patients with high levels of LDL_C was significantly higher.
5. Regardless of gender stratification, baseline information SBP, course of disease (time), laboratory test index SCR, and complication index DN were risk factors for DR.
6. Baseline data were BMI (continuous), BMI (classification), and DBP; laboratory test indicators were HbA1c, TG, UA, Val, eGFR (continuous), eGFR (classification), and complication indicators: CHD and stroke were not statistically different from DR regardless of gender stratification.

**Table 1:** Difference analysis of related indicators of DR after gender stratification.

Variables	Women (558)		p	Men (591)		p
	DR (143)	N-DR (415)		DR (126)	N-DR (465)	
	Median/N (q1–q3/%)	Median/N (q1–q3/%)		Median/N (q1–q3/%)	Median/N (q1–q3/%)	
age	63.00 (56.00–69.00)	62.00 (54.00–69.00)	0.514	60.00 (51.00–66.00)	56.00 (47.00–65.00)	0.019
age1 = 1	61 (42.66%)	175 (42.17%)	0.919	39 (30.95%)	121 (26.02%)	0.269
time	14.00 (7.00–20.00)	6.00 (1.00–12.00)	<0.0001	13.50 (6.00–20.00)	6.00 (1.00–10.00)	<0.0001
time1 = 1	36 (25.17%)	232 (55.90%)		40 (31.75%)	265 (56.99%)	
time1 = 2	66 (46.15%)	147 (35.42%)		53 (42.06%)	164 (35.27%)	
time1 = 3	41 (28.67%)	36 (8.67%)		33 (26.19%)	36 (7.74%)	
smoke = 1	4 (2.80%)	20 (4.82%)	0.304	47 (37.30%)	207 (44.52%)	0.147
drink = 1	2 (1.40%)	9 (2.17%)	0.568	32 (25.40%)	159 (34.19%)	0.061
BMI	25.71 (23.05–28.31)	25.34 (22.89–28.44)	0.653	26.11 (24.09–28.40)	26.12 (24.06–28.40)	0.846
BMI1 = 1	1 (0.70%)	7 (1.69%)	0.837	1 (0.79%)	7 (1.51%)	0.918
BMI1 = 2	46 (32.17%)	135 (32.53%)		29 (23.02%)	107 (23.01%)	
BMI1 = 3	58 (40.56%)	160 (38.55%)		58 (46.03%)	219 (47.10%)	
BMI1 = 4	38 (26.57%)	113 (27.23%)		38 (30.16%)	132 (28.39%)	
SBP	150.00 (132.00–167.00)	144.00 (129.00–158.00)	0.016	147.00 (131.00–159.00)	141.00 (127.00–155.00)	0.013
DBP	78.00 (71.00–87.00)	80.00 (72.00–88.00)	0.638	80.50 (75.00–89.00)	83.00 (76.00–91.00)	0.138
leu	112.44 (93.79–130.85)	115.27 (97.26–136.46)	0.215	122.62 (102.47–144.01)	130.66 (108.33–158.57)	0.003
leu1 = 1	54 (37.76%)	179 (43.13%)	0.261	67 (53.17%)	289 (62.15%)	0.068
Val	135.53 (114.13–161.24)	131.96 (113.11–160.91)	0.506	143.08 (121.54–165.85)	145.42 (121.21–171.59)	0.616
HbA1c	9.00 (7.40–10.60)	8.60 (7.40–10.50)	0.563	8.55 (7.20–10.60)	8.90 (7.30–10.50)	0.633
SCR	45.24 (3.69–58.01)	35.66 (2.87–51.48)	0.000	62.82 (5.34–83.13)	41.80 (2.75–70.04)	<0.0001
TG	1.63 (1.04–2.48)	1.69 (1.17–2.35)	0.355	1.56 (1.08–2.86)	1.62 (1.11–2.46)	0.775
UA	305.00 (242.09–349.00)	297.00 (238.00–354.12)	0.580	357.61 (295.00–440.64)	345.00 (283.60–403.35)	0.054
HDL_C	1.40 (1.18–4.16)	2.12 (1.25–4.77)	0.012	1.22 (0.97–2.79)	2.02 (1.11–4.44)	0.000
HDL_C1 = 1	38 (26.57%)	88 (21.20%)	0.072	57 (45.24%)	157 (33.76%)	0.007
HDL_C1 = 2	72 (50.35%)	190 (45.78%)		50 (39.68%)	180 (38.71%)	
HDL_C1 = 3	33 (23.08%)	137 (33.01%)		19 (15.08%)	128 (27.53%)	
LDL_C	2.12 (1.33–2.78)	1.57 (1.09–2.58)	0.008	2.03 (1.22–2.73)	1.44 (0.93–2.50)	0.001
LDL_C1 = 1	68 (47.55%)	250 (60.24%)	0.029	62 (49.21%)	297 (63.87%)	0.008
LDL_C1 = 2	41 (28.67%)	87 (20.96%)		40 (31.75%)	95 (20.43%)	
LDL_C1 = 3	34 (23.78%)	78 (18.80%)		24 (19.05%)	73 (15.70%)	
eGFR	90.00 (53.00–90.00)	83.40 (50.31–90.00)	0.280	90.00 (67.91–90.00)	86.38 (63.70–90.00)	0.327
eGFR1 = 1	44 (30.77%)	154 (37.11%)	0.392	24 (19.05%)	82 (17.63%)	0.195
eGFR1 = 2	22 (15.38%)	59 (14.22%)		34 (26.98%)	165 (35.48%)	
eGFR1 = 3	77 (53.85%)	202 (48.67%)		68 (53.97%)	218 (46.88%)	
DN = 1	87 (60.84%)	116 (27.95%)	<0.0001	72 (57.14%)	119 (25.59%)	<0.0001
CHD = 1	21 (14.69%)	72 (17.35%)	0.461	15 (11.90%)	69 (14.84%)	0.403
stroke = 1	12 (8.39%)	46 (11.08%)	0.363	11 (8.73%)	60 (12.90%)	0.201

After normality test, all continuous variables do not obey normal distribution. Median (quartile spacing) is used for statistical description, and rank-sum test is used to analyze whether there are differences. Categorical variables were described statistically using numbers (percentage) and chi-square test.

BMI, body mass index; SBP, systolic blood pressure; DBP, diastolic blood pressure; HbA1c, glycosylated hemoglobin; SCR, serum creatinine; TG, triglyceride; UA, urinary creatinine; HDL_C, high-density lipoprotein cholesterol; LDL_C, density lipoprotein cholesterol; eGFR, estimated glomerular filtration rate; DN, diabetic nephropathy; CHD, coronary heart disease.

### The Relationship Between Leucine and Diabetic Retinopathy

**Figure 1:**
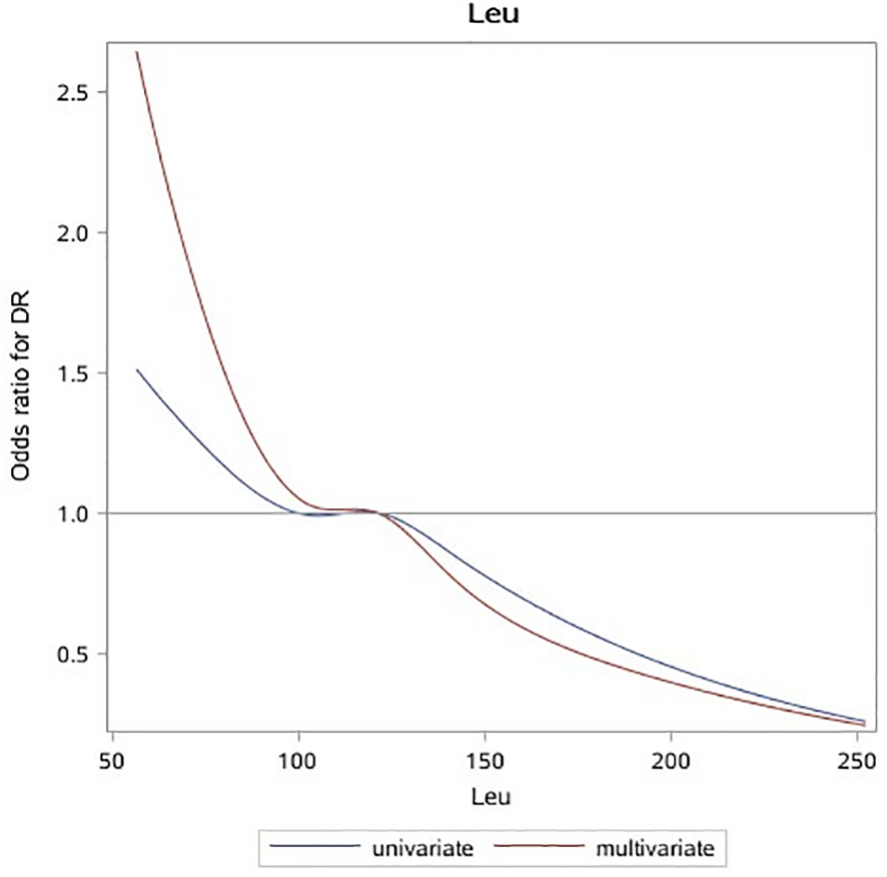
OR curves of leu and DR in patients with T2D. The blue solid line represents the curve with only leu in the model, and the red dotted line represents the curve after adjusting for all baseline data, laboratory test indicators, and complications. It can be seen that leu is a protective factor of DR. OR, odds ratio; leu, leucine; DR, diabetic retinopathy; T2D, type 2 diabetes.

After processing leu with RCS, it was found that the OR curve of the occurrence probability of leu and DR met a linear relationship, and the risk would gradually decrease with the increase of leu. As can be seen from the observation in **Figure 1**, OR can be obviously divided into two categories with 1 as the cutoff point. In this paper, the last point with OR equal to 1 was taken as the cutoff point, i.e., 120 μmol/L, which was divided into two categories (**Figure 1**). In this paper, continuous variables in the covariables were processed according to whether they met the linear relationship with the occurrence probability of DR. Among them, age, course of the disease, HDL_C, LDL_C, and eGFR, which did not meet the linear relationship, were classified according to the method previously mentioned and were included in the model as categorical variables. The remaining continuous variables satisfy linearity and are incorporated into the model as continuous variables.

In univariate logistic regression (Model1), leu was a protective factor of DR when included as a continuous variable (OR: 0.78 (0.66, 0.92)), and the difference was not statistically significant after classification. After adjusting covariables such as baseline information and laboratory tests, the results were the same as single factor regression, and the protective effect of leu decreased, among which the changes were more obvious after adjusting laboratory indicators (Model2: (OR: 0.79 (0.65, 0.95)), Model3: (OR: 0.81 (0.67, 0.99))). There was no statistically significant difference between the incidence of leu and DR after the inclusion of complications (Model4), regardless of whether leu was included as a continuous or categorical variable (**Table 2**).

**Table 2:** Risk OR of leu and DR under multiple models.

	OR (95% CI)	p
Univariable Model1		
Leu per μmol/L	0.78 (0.66, 0.92)	0.004
<120	0.76 (0.56, 1.04)	0.085
>120		
Multivariable Model2		
Leu per μmol/L	0.79 (0.65, 0.95)	0.014
<120	0.77 (0.55, 1.08)	0.134
>120		
Multivariable Model3		
Leu per μmol/L	0.81 (0.67, 0.99)	0.037
<120	0.82 (0.58, 1.16)	0.259
>120		
Multivariable Model4		
Leu per μmol/L	0.83 (0.68, 1.02)	0.081
<120	0.88 (0.62, 1.25)	0.481
>120		

leu was incorporated into the model according to classification variables and continuous variables (2 categories). Leu(Model1), leu + baseline data (Model2), leu + baseline data + laboratory test index (Model3), leu + baseline data + laboratory test index + complication index (Model4).

OR, odds ratio; DR, diabetic retinopathy; leu, leucine.

### Interaction Between Leucine and Gender

**Figure 2:**
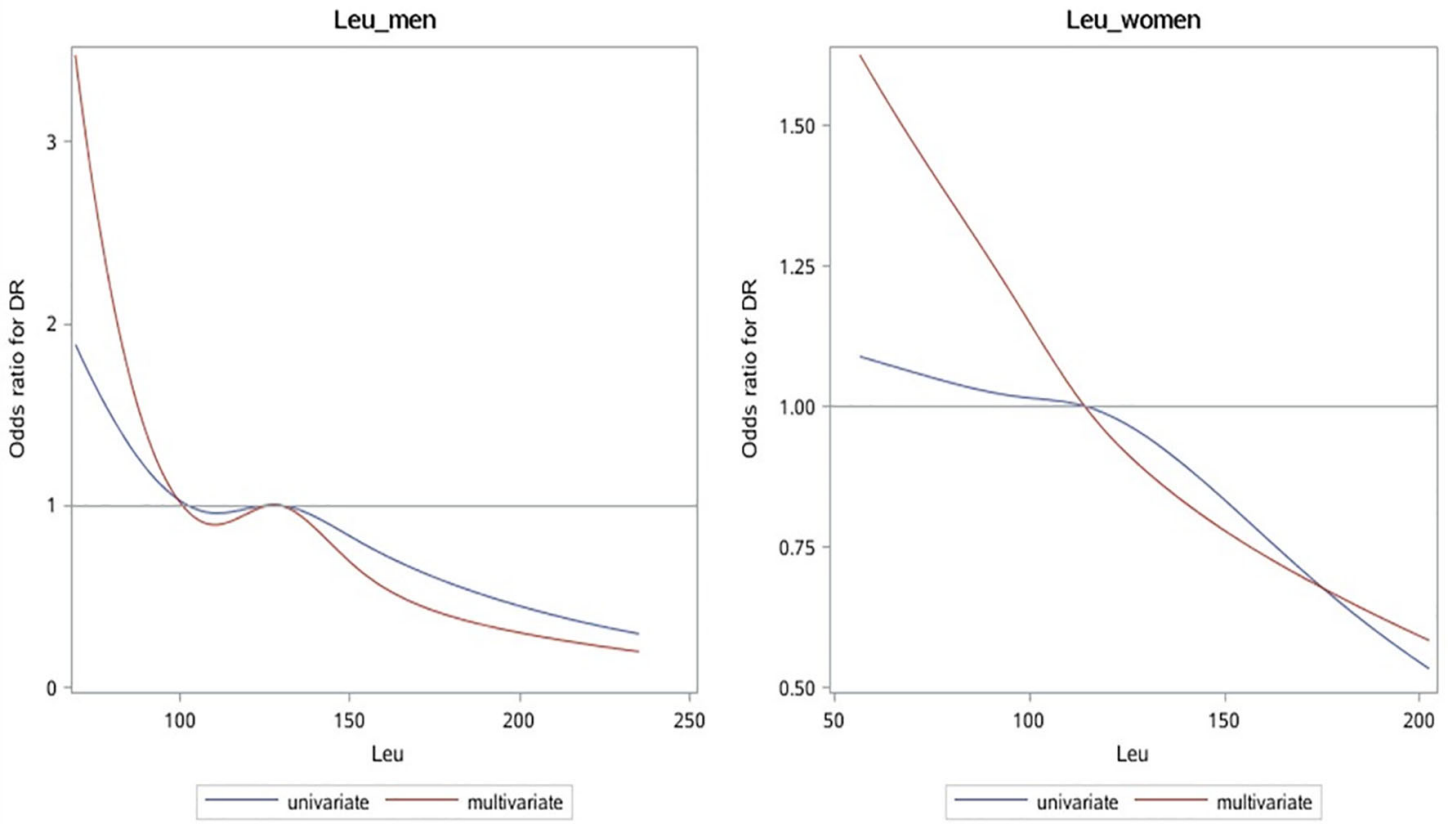
OR curves of leu and DR in patients with T2D of different genders. The blue solid line represents the curve with only leu in the model, and the red dotted line represents the curve after adjusting for all baseline data, laboratory test indicators, and complications. It can be seen that leu is a protective factor of DR. OR, odds ratio; leu, leucine; DR, diabetic retinopathy; T2D, type 2 diabetes.

The correlation between leu and DR risk changed in different genders (**Figure 2**, **Table 3**). OR shows that leu is the protective factor of DR in all models, but p-value shows that in the condition of 0.05, only when leu is included as a continuous variable, in the single-factor model (Model1) and multi-factor model (Model2), leu was statistically significant as a protective factor for DR in men (Model1 (OR: 0.73 (0.58, 0.94)); Model2 (0.77 (0.59, 1.00))).

**Table 3:** OR of leu and DR under multiple models of different genders.

	Female (558)		Male (591)	
	OR (95% CI)	p	OR (95% CI)	p
Univariable Model1				
Leu per μmol/L	0.87 (0.68, 1.12)	0.282	0.73 (0.58, 0.94)	0.013
<120	0.83 (0.54, 1.28)	0.399	0.78 (0.49, 1.22)	0.274
>120				
Multivariable Model2				
Leu per μmol/L	0.83 (0.62, 1.10)	0.187	0.77 (0.59, 1.00)	0.046
<120	0.72 (0.45, 1.15)	0.168	0.85 (0.52, 1.38)	0.500
>120				
Multivariable Model3				
Leu per μmol/L	0.84 (0.63, 1.12)	0.240	0.78 (0.59, 1.04)	0.087
<120	0.74 (0.46, 1.20)	0.220	0.92 (0.55, 1.53)	0.742
>120				
Multivariable Model4				
Leu per μmol/L	0.86 (0.64, 1.16)	0.332	0.80 (0.60, 1.07)	0.137
<120	0.76 (0.46, 1.24)	0.275	1.01 (0.60, 1.72)	0.969
>120				

Stratified the data by gender, and leu was incorporated into the model according to classification variables and continuous variables (2 categories). Leu(Model1), leu + baseline data (Model2), leu + baseline data + laboratory test index (Model3), leu + baseline data + laboratory test index + complication index (Model4).

OR, odds ratio; DR, diabetic retinopathy.

The calculated additive interaction between gender and leu (classification variable) are RERI 0.20 (−0.33, 0.73), AP 0.28 (−0.04, 0.59), and S 0.57 (0.16, 2.10); the results suggest that there is no interaction between gender and leu.

### Sensitivity Analysis

**Figure 3:**
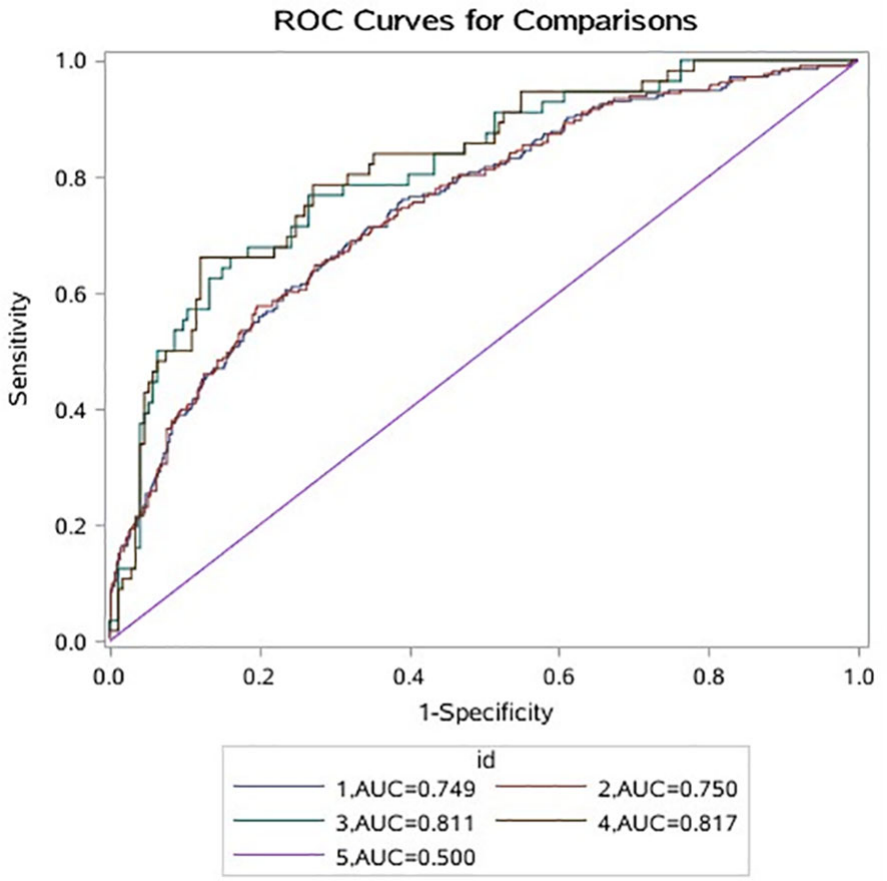
1 indicates that A data do not contain the ROC curve corresponding to leu; 2 indicates that A data contain the ROC curve corresponding to leu; 3 indicates that B data do not contain the ROC curve corresponding to leu; 4 means that B data contain ROC curve corresponding to leu; 5 represents ROC invalid curve. ROC, receiver operating characteristic.

Of the data, 80% are used as training set A, and the remaining 20% are used as verification set B. The results of training set A and verification set B are consistent, the area under the curve (AUC) is significantly greater than 0.500, indicating that the model is valid. The AUC of the model containing leu is larger than that of the model without leu (training set A: (AUC: 0.750 vs. 0.749); validation set B: ((AUC: 0.817 vs. 0.811)); the model was better when leu was included (**Figure 3**).

## Discussion

This study found that the content of leu in the DR population was lower than that in the N-DR population, which was a protective factor of DR, consistent with the previous research results (41–46). Literature (33, 34, 45, 46) on the study of diabetes and its complications has concluded that improved glycemic control is effective in reducing the risk of microvascular and macrovascular complications and cardiovascular disease. Based on this, we suspect that the protective effect of leu on DR is likely to be by the same mechanism as its protective effect on diabetic patients. It has been found that leu is a proinsulin agent both *in vivo* and *in vitro* by activating mTOR complex 1 (mTORC1), which both induces and enhances insulin secretion from pancreatic beta cells and also helps to maintain beta-cell mass (47). After stratification by gender, leu remained a protective factor for DR in men, but the difference was not statistically significant in women. The above differences may be caused by differences in dietary habits of different genders (48–50). The data in this paper show that in both DR and N-DR populations, the content of leu in the male population is significantly higher than that in the female population. Leu is an essential amino acid that can only be obtained from the diet, which is related to men’s preference for a high-protein diet (50). It is suggested that leu can regulate insulin metabolism only after basic physiological requirements are satisfied. Our previous studies found that the leu in whole gender group and women are protective factors, the above phenomenon was not observed in men (51), and the same finding that men more than women tend to eat a high-protein diet, while in the form of food protein, intake of leu increases other kidney burdens of metabolic components at the same time, to offset the leu protective effect in men. This conclusion suggests that the human body can appropriately supplement high-protein food, but a long-term high-protein diet may increase the burden of nephropathy in T2D patients (52) and promote the occurrence of DN (53). The patient’s healthcare and treatment should still follow the doctor’s advice and be reviewed regularly.

The results of the multivariate regression model showed that leu was a protective factor of DR in all gender populations, OR was 0.78 (0.66, 0.92), and the difference was statistically significant. After gender stratification, the OR was lower than 1 in all women and most men. Although the statistical results showed that the value of OR was not statistically significant after gender stratification, the single-point OR value still suggested that DR might be a protective factor of DR. It is well known that the control of blood glucose in diabetic patients can effectively reduce the occurrence of microvascular complications (DR and DN) (54, 55). Meanwhile, a number of studies (56, 57) have found that the abnormal activation of mTORC1 in the central insulin signaling pathway in T2D patients can cause insulin resistance. Leu can significantly improve the excitability of mTOR (28, 58, 59), which also proves to some extent that leu is a protective factor for DR. It is speculated that leu can be used as a potential predictor of DR, and it is necessary to construct different application criteria for different genders.

This paper has some limitations: 1) this paper only collected the data of the Second Affiliated Hospital of Dalian Medical University and the First Affiliated Hospital of Jinzhou Medical University, which are concentrated in Liaoning Province and have obvious regional characteristics in climate, diet, humanities, and other aspects, which is not conducive to promoting the application to the whole country. It is recommended to be used in northeast China, and we will cooperate to collect national data for analysis in the future. 2) In the data, smoking and drinking were only collected as yes or no, but not how long their duration was. As a result, it is concluded in this paper that smoking and drinking are the protective factors of DR, which is not consistent with common cognition. Confounding factors, such as whether the above relationship is real or how long it was, need to be collected for more detailed data to verify the above relationship. 3) This paper is a cross-sectional study, and its ability as evidence is weaker than that of the cohort. If we have the ability to conduct cohort studies in the future, we hope to increase the credibility of the results. 4) We did not grade the severity of DR and may not be able to distinguish the differences that exist between the different severity levels.

## Conclusion

In all-gender population and male patients, leu was a protective factor of DR regardless of whether it was included in the model in the form of a continuous variable or categorical variable. These relationships were not statistically significant in women. It is suggested that leu may be a new direction predicted by DR, but it needs to be applied by gender. Regardless of disease (DR or DN), leu in men is higher than that in women, suggesting that leu may regulate insulin metabolism on the premise of meeting basic physiological needs.

## Data Availability Statement

The raw data supporting the conclusions of this article will be made available by the authors, without undue reservation.

## Ethics Statement

The studies involving human participants were reviewed and approved by the Second Affiliated Hospital of Dalian Medical University the First Affiliated Hospital of Jinzhou Medical University. The patients/participants provided their written informed consent to participate in this study.

## Author Contributions

All authors have participated extensively in the study and had proofread the final manuscript. SL and BH conceived and designed the research. BH performed data cleaning and data analysis. SL, BH, M-LL, X-TC, Y-FC, and Z-NG participated in the interpretation and discussion of the results. SL and BH wrote the manuscript. Y-FC and Z-NG approved and reviewed the final manuscript. All authors read and approved the final manuscript.

## Funding

This research was financially supported by the development and application of clinical mass spectrometry detection technology for important small-molecule metabolites (2020JH2/10300116), Key R&D Program of Liaoning Province, and Platform Name: Dalian Laboratory Medicine Mass Spectrometry Technology Innovation Center.

## Conflict of Interest

Authors BH, M-LL, and X-TC were employed by the company Dalian Runsheng Kangtai Medical Lab Co. Ltd. Author Y-FC was employed by Shanghai Engineering Research Center of Reproductive Health Drug and Devices.

The remaining authors declare that the research was conducted in the absence of any commercial or financial relationships that could be construed as a potential conflict of interest.

## Publisher’s Note

All claims expressed in this article are solely those of the authors and do not necessarily represent those of their affiliated organizations, or those of the publisher, the editors and the reviewers. Any product that may be evaluated in this article, or claim that may be made by its manufacturer, is not guaranteed or endorsed by the publisher.
